# Relevant markers for overactive bladder laser therapy: nitric oxide and urinary nerve growth factor

**DOI:** 10.1007/s10103-025-04330-0

**Published:** 2025-02-06

**Authors:** Rasha Ahmed, Omnia Hamdy, Mona Mohamed Abdulwehab, Ibrahim Abdel-Halim, Amany Ahmed Soliman, Shaimaa Elattar

**Affiliations:** 1 Urology Department, Faculty of Medicine for Girls, Al Azhar University, Cairo, Egypt; 2 Engineering Applications of Lasers Department, National Institute of Laser Enhanced Sciences, Cairo University, Giza, Egypt; 3 Clinical Pathology Department, Faculty of Medicine for Girls, Al Azhar University, Giza, Egypt

**Keywords:** Photobiomodulation, Low-level lasers, Overactive bladder, Nitric oxide, Urinary nerve growth factor

## Abstract

To investigate the potential of nitric oxide (NO) and urinary nerve growth factor (NGF) as indicators of therapeutic outcomes in overactive bladder (OAB) patients undergoing low-level laser therapy (LLLT) via a prospective randomized controlled trial. Fifty OAB patients participated in the study and were subjected to LLLT using 650-nm laser irradiation. The study employed a prospective randomized controlled trial design. Nitric oxide and urine NGF levels were assessed before and after the LLLT intervention to evaluate their correlation with therapeutic outcomes. The study provided evidence supporting the effectiveness of LLLT as a treatment modality for OAB. Analysis of NO and urine NGF levels revealed significant changes following LLLT intervention suggesting their potential as biomarkers for assessing therapeutic response in OAB patients. These biomarkers hold promise for aiding clinicians in evaluating treatment response and personalizing therapy approaches for OAB patients. This study highlights the utility of LLLT in managing OAB and underscores the importance of exploring biomarkers such as nitric oxide and urinary nerve growth factor to enhance treatment efficacy assessment. The findings suggest that NO and urine NGF levels may serve as valuable indicators of therapeutic outcomes in OAB patients undergoing LLLT. Further research is warranted to elucidate the underlying mechanisms and optimize the clinical application of LLLT in OAB management.

## Introduction

Overactive bladder (OAB) presents as a multifaceted clinical syndrome characterized by symptoms such as urine urgency, frequency, and nocturia, often accompanied by urgency urinary incontinence. Studies indicate an overall prevalence of 16.6% in European individuals aged over 40, with an increase in prevalence observed with advancing age. The exact pathophysiology of OAB remains elusive, potentially involving a complex interplay of various factors [1]. The etiology of hyperactive bladder remains unclear, although certain risk factors such as obesity, constipation, and heightened coffee consumption have been identified [2]. Additionally, conditions like limited functional mobility, poorly managed diabetes, and chronic pelvic pain may exacerbate symptoms [3]. Individuals often endure these symptoms for an extended duration before seeking medical attention, with caregivers occasionally being the ones to recognize the condition [4].

The patient’s symptoms and indicators are used to make the diagnosis; other conditions such as urinary tract infections or neurological diseases must be taken into account. Pee only comes out in little amounts during urination. In certain situations, urodynamic investigations may be utilized to diagnose pain during urination which indicates the presence of a problem other than hyperactive bladder [5]. Getting a specific treatment isn’t always required. When seeking treatment, the first recommendations are for pelvic floor exercises, bladder training, and other behavioral strategies [6]. It can be beneficial to cut back on caffeine, help overweight people lose weight, and take anti-muscarinic medications, b3 agonists, and moderate amounts of fluids. Direct injection of botulinum toxin into the bladder is an additional option. A few non-invasive electrical stimulation treatments appear to be effective when used [7].

Low-level laser treatment, also known as photobiomodulation, applies low-power lasers or light-emitting diodes (LEDs) to the skin or other body surfaces in order to enhance cell activity or lessen discomfort [8, 9]. Furthermore, fibroblasts which are the cells that comprise connective tissue are believed to be affected in a way that accelerates the healing process and has anti-inflammatory qualities [10]. A specific range of wavelengths appears to be the extent of the effects and administering LLLT below the dosage range doesn’t seem to provide any advantages [11, 12]. Based on the established effectiveness of low-level laser therapy (LLLT) in alleviating pain arising from uterine smooth muscle contractions, we posited that it could potentially offer relief for overactive bladder (OAB) symptoms resulting from involuntary muscle contractions in the bladder. Previous studies have indicated the presence of light receptors in smooth muscle cells supporting the rationale for investigating the potential efficacy of LLLT in OAB management [13].

LLLT has become known for its ability to reduce pain and inflammation while also promoting wound healing [14]. When photoreceptor molecules in the mitochondria are activated, levels of adenosine triphosphate and reactive oxygen species increase [15, 16]. This is followed by the activation of transcription factors that produce pro-proliferation, anti-oxidant, and anti-apoptotic gene products. Enhanced ATP production from LLLT also boosts nitric oxide creation (a potent vasodilator that promotes enhanced blood flow) resulting in nutrition delivery to the stimulated areas [17]. Nitric oxide (NO) is induced by LLLT and causes smooth muscle relaxation [18].

Nerve growth factor (NGF) is a tiny secreted protein that promotes the growth and survival of certain target neurons. In the urinary bladder, NGF is generated by intracellular routes regulated by protein kinase C and A. These pathways have physiological and pathological consequences in the lower urinary tract [19, 20]. NGF has been linked to the stimulation of afferent neurons, resulting in bladder hyperactivity [21]. Additionally, NGF has been associated with mechanical stretch and reflex bladder action [22]. Sensory neurons may absorb NGF and transfer it retrogradely through the central nervous system. Consequently, NGF synthesis may be a biomarker for neuroplasticity via a common process involved in the aetiology of OAB. The primary objective of this study is to investigate the potential role NO and NGF as indicators of therapeutic outcomes in patients with OAB undergoing LLLT.

## Materials and methods

### Subjects

A single-center, randomized, prospective, comparative study was undertaken involving a cohort of 50 female patients, aged 23 to 61 years, who sought treatment at the outpatient clinic of the urology department at Al-Zahraa University Hospital. Patients enrolled in the study presented with symptom duration of one year and exhibited bladder overactivity as assessed by urodynamic cystometry study and exhibited also mild to moderate overactive bladder symptoms, as assessed by standardized symptom scoring criteria. The OABSS is a simplified version of the questionnaire that was developed in 2006 [23]. The main concept of OABSS is based on core symptoms of OAB based on the ICS definition [24] urinary urgency, usually with urinary frequency and nocturia, with or without UUI. The severity of OAB symptoms is commonly classified according to the OABSS score —mild (≤ 5), moderate (6–11), and severe (≥ 12) [25]. Participation in the study was not permitted for patients who had a history of pelvic irradiation, infra-vesical symptom obstruction, urinary infection, lower ureteric stone, uncooperativeness, epilepsy, previous diagnosis of an invasive tumor, prior surgical treatment for urinary incontinence, pregnancy, or recent administration of overactive bladder (OAB) medication within three months, underactive bladder or bladder outlet obstruction proved by pressure flow study. Upon enrollment, all eligible patients received detailed information regarding the study’s objectives, procedures, and potential risks, and provided written informed consent prior to participation.

### Study design

**Fig. 1:**
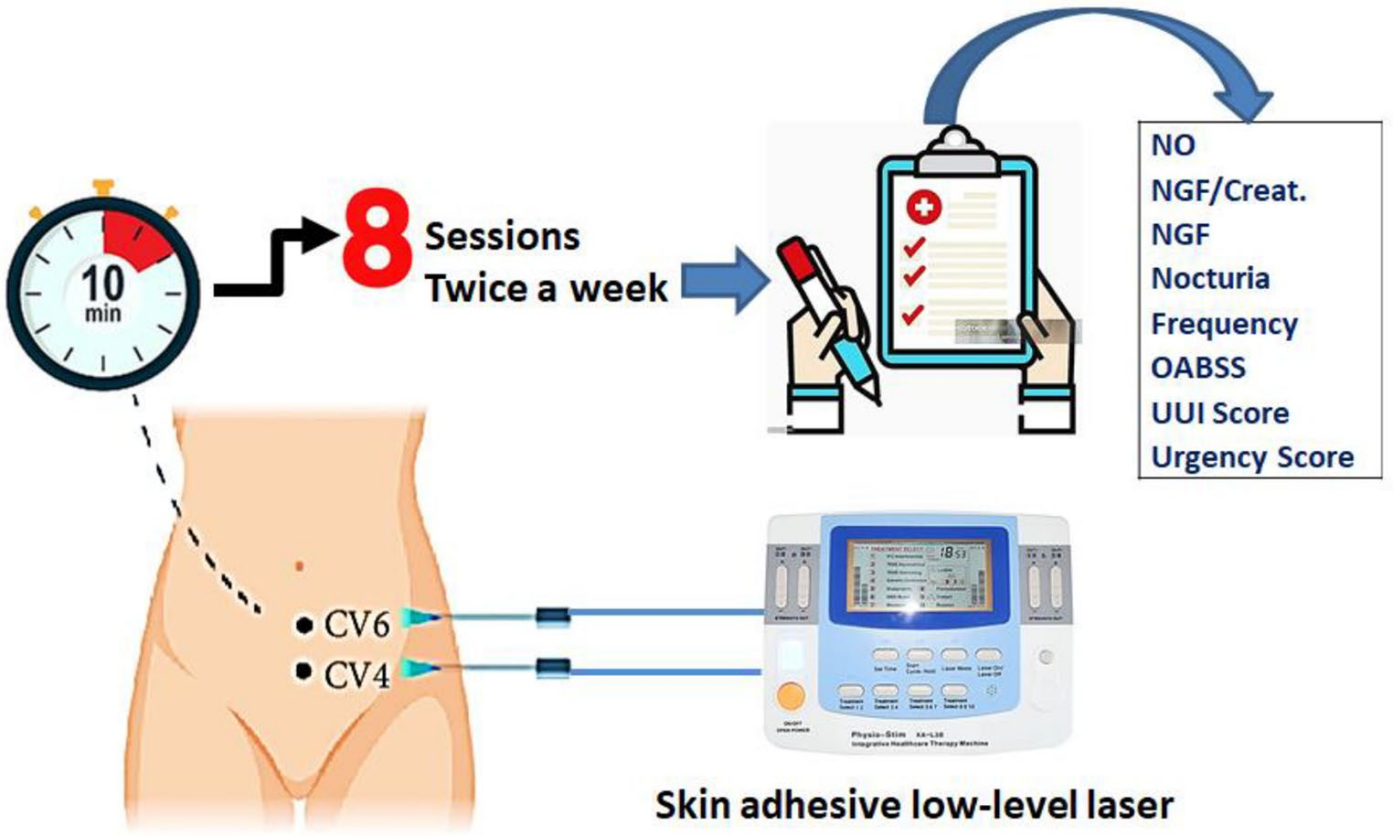
Schematic of the proposed study design and steps

Eight sessions of skin adhesive low-level laser (physio-stim, model: KA-L38, China) at 650 nm wavelength and power ≤ 5 mw were administered twice a week to each of the participants with detrusor overactivity at two aquapuncture locations, CV4 and CV6. For every point, there were roughly ten minutes in each session as illustrated in Fig. 1. The average power density (i.e. irradiance) at the target areas was 0.32 mW/cm^2^.

Based on the presumption that some acupoints (CV4, CV6) related to a meridian, the frequencies used in this investigation were in line with the Reininger Meridian Frequencies. Both acupuncture sites are thought to relieve OAB symptoms because they are crucial as targeted points for female urology [26]. Age and body mass index were recorded. Numbers and scores of urine frequency and nocturia, urgency and urge urinary incontinence scores, urodynamic cystometric parameters, OAB-questionnaire, OABSS, serum NO level, urine NGF and urinary NGF/Cr were the baseline data of the control group (before laser sessions) that were investigated. Following therapy, every patient was reassessed using the same criteria so that the results could be compared to the control group. The OAB questionnaire was used to determine whether or not a woman had bladder overactivity, and urodynamic findings were correlated with the presence or absence of OAB symptoms [27].

The selection of CV4 and CV6 as treatment points was based on their historical significance in female urology and their potential relevance in alleviating symptoms of overactive bladder. While focusing the irradiation area closer to the bladder could theoretically enhance the effects on bladder muscles, the decision to use these acupoints was rooted in their traditional therapeutic associations. Optimal targeting of the bladder muscles might involve positioning the irradiation area as close to the bladder as possible, potentially intensifying the direct therapeutic impact of LLLT on the bladder muscles and surrounding tissues. In the current study, the utilized probe compresses the pointed areas CV4 and CV6, displacing excess fluid and enhancing laser penetration to deep structures. However, considerations such as practicality and safety must be balanced to ensure the effective and safe administration of the treatment [28].

### Biomarkers measurements

Blood and urine samples were obtained from each individual prior to and following laser sessions. Furthermore, the NO level, urine NGF, and urinary creatinine (u Cr) levels were evaluated.

### Urinary NGF and creatinine measurements

Every individual provided a urine sample which was subsequently centrifuged for 20 min at 4 °C and 3,000 rpm. The 1.5 mL eppendorf tubes containing the divided aliquots of the supernatant were stored at -80 °C. The urinary NGF concentration was measured using the Human Nerve growth factor ELISA Kit (Bioassay Technology Laboratory, Zhejiang, China, Cat. No E2102Hu). An antibody against human NGF has been pre-coated onto the plate. After being introduced, the sample’s NGF binds to the antibodies coated on the wells. Next, the material is exposed to biotinylated Human NGF Antibody, which attaches itself to NGF. Following the addition of streptavidin-HRP, the biotinylated NGF antibody binds to it. During a washing step following incubation, unbound streptavidin-HRP is removed. Following the addition of the substrate solution, color changes in accordance to the concentration of human NGF. An acidic stop solution is added to stop the process. The Das Italy ELISA Microplate Reader, operating at 450 nm, was used to calculate the quantity of NGF.

The urinary creatinine was measured using Buffered jaffé reaction (Cat. No MG234 001). Before the experiment, all samples were diluted from 1 part sample to 49 parts isotonic saline. To adjust for dilution, multiply the result by 50 and measure at 492 nm with a chem-100 photometric analyzer. The Human Nitric Oxide ELISA Kit was used to assess the serum NO level. Human NO antibody has been pre-coated on the plate. The sample’s NO is introduced and attaches itself to the well-coated antibodies. Then, after being introduced, the biotinylated Human NO Antibody binds to the NO in the sample. Next, the biotinylated NO antibody binds to streptavidin-HRP. During a washing phase, unbound streptavidin-HRP is removed following incubation. After adding the substrate solution, the color changes in direct proportion to the concentration of human NO. The addition of an acidic stop solution ends the process, and the absorbance is measured at 450 nm.

### Statistical analysis

ROC is a graphical representation that is used to examine the accuracy of diagnostic tests via demonstrating the sensitivity vs. 1-specificity of a certain test or set of tests. When evaluating the efficacy of a discriminating method (test), the area under the ROC curve (AUC) is considered the most significant metric since a larger AUC indicates a higher level of accuracy in the test [29, 30]. Creating a ROC curve is based on the concepts of true positive, true negative, false positive, and false negative. The curve’s X-axis represents 1-specificity, or the false positive fraction, while the Y-axis represents sensitivity, or the genuine positive percentage [31]. The present investigation used a proprietary Matlab function in conjunction with the MATLAB software framework to construct the presented ROC curves. This function represents the 1-specificity and sensitivity of two classes of data to plot the ROC curve and compute its parameters (i.e. non-irradiated and irradiated (or laser-treated) samples in our implementation.

## Results and discussion

**Fig. 2:**
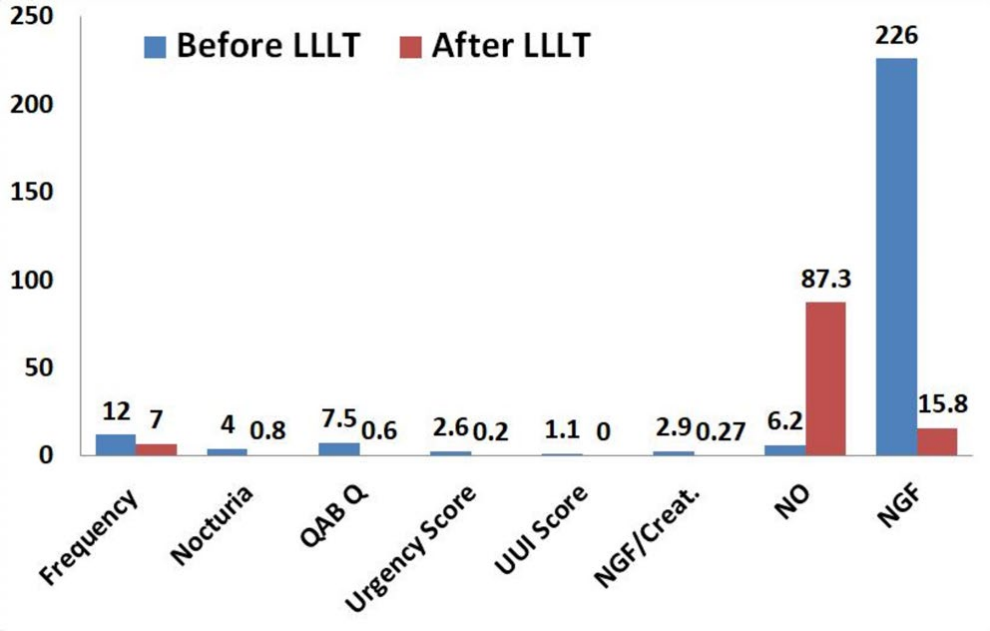
Variation in the whole studied biomarkers among participants before and after the application of the LLLT(the presented values are the mean values of the all collected medical records)

In this randomized comparative study, we investigated the efficacy of skin-adhesive LLLT in the management of OAB symptoms. A total of 50 OAB subjects were enrolled with a mean age of 43 years and a mean BMI of 28. The primary objective was to evaluate the impact of LLLT on OAB symptoms, while also exploring the potential of NGF and NO levels as biomarkers for treatment outcome assessment. In patients diagnosed with mild to moderate overactive bladder symptom severity (OABSS), the administration of LLLT using the designated device for a total of 8 treatment sessions resulted in a statistically significant reduction in the frequency and nocturia episodes (see Fig. 2). The mean frequency decreased to 7 (range: 5–10) and the mean nocturia episodes reduced to 0.8 (range: 0–2), as compared to the control group with a mean frequency of 12 (range: 10–14) and mean nocturia episodes of 4 (range: 2–6). Furthermore, there was a significant decrease in urgency scores and the number of episodes of urgency urinary incontinence (UUI) which are considered key symptoms of OAB. The urgency scores ranged from 0 to 1 and there were no UUI episodes reported, whereas the control group exhibited urgency scores ranging from 1 to 5 and UUI episodes ranging from 1 to 3.

**Fig. 3:**
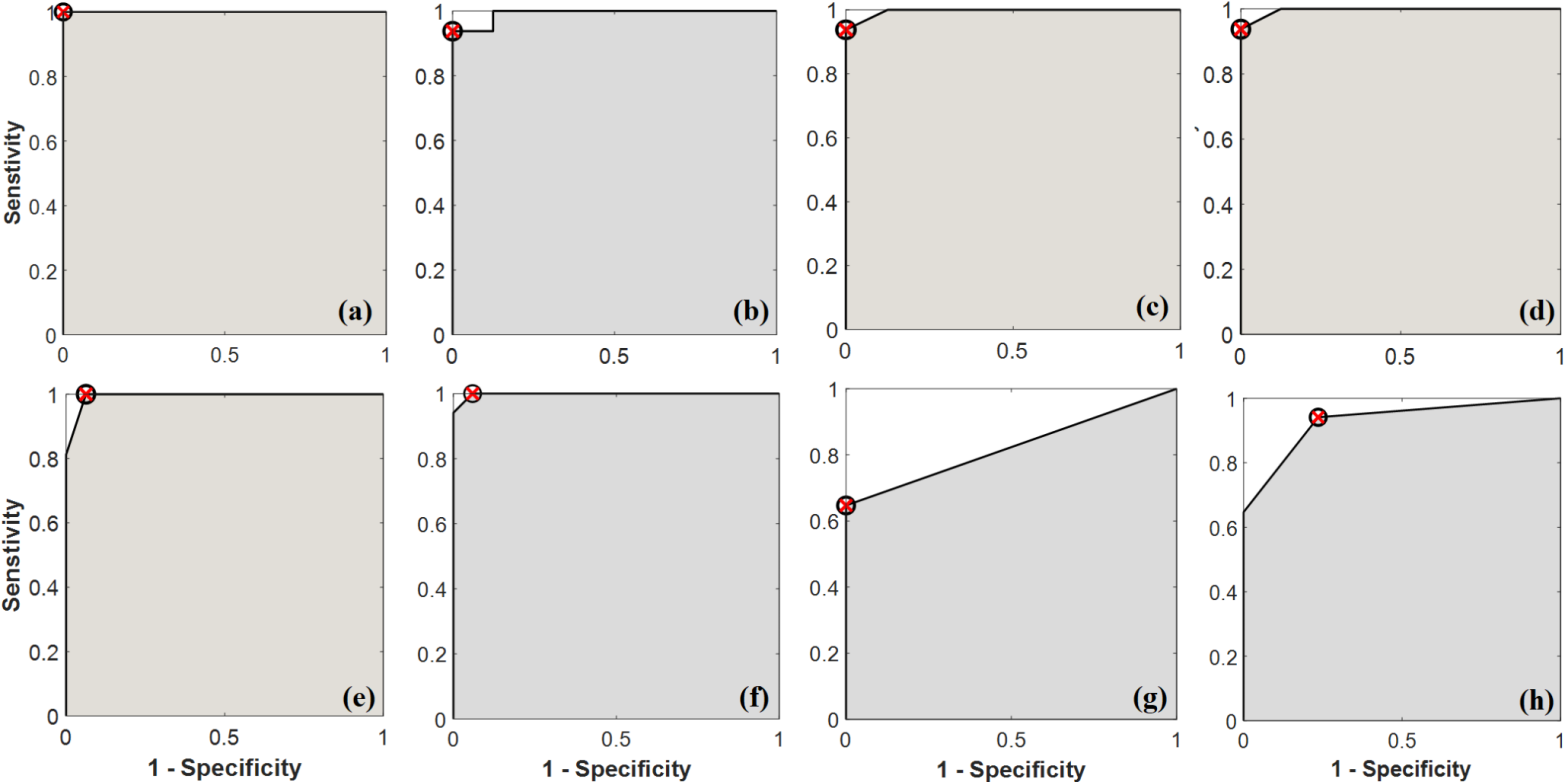
ROC curves for each marker (**a**) NO, (**b**) NGF/Creat., (**c**) NGF, (**d**) Nocturia, (**e**) Frequency, (**f**) OABSS, (**g**) UUI Score, (**h**) Urgency Score

The ROC curve analysis was performed to assess the discriminatory ability of urinary overactive bladder symptoms (frequency, nocturia, urgency, and urgency urinary incontinence [UUI]) before laser treatment compared to the symptoms at the completion of treatment. The results demonstrated a significant reduction in the number of frequency and nocturia episodes. The sensitivity and specificity values for frequency were 1.000 and 0.973 respectively, and for nocturia, the sensitivity and specificity values were 0.937 and 1.000 respectively. The accuracy values were 0.968 for frequency and 0.969 for nocturia. The AUC values were 0.994 for frequency and 0.996 for nocturia. Furthermore, there was a significant improvement in urgency and UUI scores. The sensitivity and specificity values for urgency were 0.941 and 0.765, respectively, while for UUI, the sensitivity and specificity values were 0.700 and 1.000, respectively. The accuracy values were 0.853 for urgency and 0.824 for UUI. The AUC values were 0.929 for urgency and 0.824 for UUI. The threshold values were 0.5 for both urgency and UUI. The whole results of the ROC curve analysis are summarized in Fig. 3; Table 1.

Our results demonstrated a substantial difference in the OABSS before and after laser treatments. The sensitivity value was 1.000 indicating that the laser treatment correctly identified all individuals with OABSS improvement. The specificity value was 0.941 indicating that the laser treatment accurately identified individuals without OABSS improvement. The overall accuracy of the laser treatment in predicting OABSS improvement was 0.971. The AUC value was 0.998 indicating a high discriminatory ability of the laser treatment in distinguishing between pre- and post-treatment OABSS. The threshold value was determined to be 3.5 suggesting that individuals with an OABSS reduction of 3.5 or more were more likely to have benefited from the laser treatment.

**Table 1:** ROC curve parameters for each evaluation parameters

Parameter	Sensitivity	Specificity	Accuracy	AUC	Threshold
NO	1.000	1.000	1.000	1.000	25.29
NGF/Creat.	0.937	1.000	0.968	0.992	0.820
NGF	1.000	1.000	1.000	1.000	64.40
Nocturia	0.937	1.000	0.969	0.996	2.500
Frequency	1.000	0.973	0.968	0.994	9.000
OABSS	1.000	0.941	0.971	0.998	3.500
UUI Score	0.700	1.000	0.824	0.824	0.500
Urgency Score	0.941	0.765	0.853	0.929	0.500

Our study’s results regarding the reduction in the number of urgency and UUI episodes align with the results reported by Woo Yeon Hwang et al. [28] who observed a statistically significant decrease in the number of urgency and UUI episodes compared to their sham group. However, there were some differences in the outcomes related to frequency, nocturia, and OABSS improvement.

It has been suggested that increased NGF levels in urine may augment bladder sensory input reaching the central nervous system and sensitize bladder afferent pathways, which may ultimately result in DO. Patients with OAB had higher levels of NGF in their urine, which lends credence to this explanation [32]. Urinary NGF levels in patients with OAB are much greater (by around 12 times) than in normal controls, according to pilot clinical trials [33]. Patients with OAB have been observed to have higher urine NGF concentrations, especially when they are complaining of urgency urinary incontinence (OAB wet) [34]. We examined the potential of urinary NGF as a biomarker for predicting the efficacy of low-level laser treatment in this study. In general, the results indicated that the LLLT group had lower levels of NGF than the control group, suggesting a relationship between this biomarker and the therapeutic response to LLLT. Based on ROC analysis, we found a significant difference in urine NGF levels between the control group and the post-LLLT group, with 1 AUC and 100% sensitivity, specificity, and accuracy.

There are two distinct isoforms of nitric oxide synthase (NOS), namely neuronal NOS (nNOS) and endothelial NOS (eNOS) are constitutively expressed in the lower urinary tract. However, nNOS is predominantly expressed in the bladder neck and urethra, while eNOS is more prevalent in the bladder. NO serves as a modulator of afferent neuron activity within the bladder. Under pathological conditions, inducible NOS expression leads to increased detrusor contractility and bladder wall thickness. Additionally, eNOS facilitates the invasion of Escherichia coli into the bladder wall, thereby contributing to recurrent urinary tract infections. In the urethra, NO plays a vital role in relaxing smooth muscle cells [35]. LLLT has been shown to enhance the production of NO. This increased NO generation acts as a potent vasodilator, promoting improved blood flow and facilitating the delivery of essential nutrients to the stimulated areas. This effect is attributed to the heightened ATP production resulting from LLLT treatment [17]. In this study, our objective was to explore the potential of serum NO levels as a biomarker for predicting the efficacy of LLLT. We observed that the LLLT group exhibited significantly higher levels of serum NO compared to the control group suggesting a correlation between this biomarker and the therapeutic response to LLLT. Through ROC analysis, we identified a substantial difference in serum NO levels between the control group and the post-LLLT group. The sensitivity was 100% indicating that the LLLT effectively identified all individuals with elevated serum NO levels. The specificity value was also 100% indicating that the LLLT accurately identified individuals without elevated serum NO levels. To the best of our knowledge, this is the first study to investigate the levels of NO and NGF in patients with overactive bladder who underwent LLLT.

In general, the proposed results demonstrated a significant improvement in OAB symptoms when comparing the LLLT group to the control group. The LLLT group not only exhibited a rise in maximal voided volume but also a decrease in urgency, frequency, and nocturia. Moreover, following therapy, it was shown that the urine samples from the LLLT group included significantly different levels of NO and NGF. The LLLT group had elevated NO and decreased NGF levels relative to the control group, which could suggest a relationship between these biomarkers and the therapeutic response to LLLT. Although this work adds to our understanding of the therapeutic effects of LLLT on OAB, more research is necessary to determine the exact processes underlying the observed alterations in NGF and NO levels. Long-term follow-up studies are also required to see whether the therapeutic effects are durable and whether LLLT has the potential to lessen the need for invasive procedures or pharmaceutical therapies.

While the current study demonstrates promising results for LLLT’s effectiveness in reducing OAB symptoms such as urgency, frequency, and nocturia, there are several areas for further investigation. Future research should focus on exploring additional parameters to further validate and expand upon these findings. One key area of investigation is the dose-response relationship between light intensity and clinical outcomes. Testing various light intensities could help determine whether higher or lower intensities yield better therapeutic efficacy. This will provide deeper insights into optimizing LLLT protocols for OAB management.

## Conclusion

In conclusion, the findings of this investigation provide insight on the potential mechanisms underlying the therapeutic effects of LLLT in the treatment of overactive bladder. The results show that LLLT dramatically reduces OAB symptoms including urgency, frequency, and nocturia. Furthermore, the study emphasizes the significance of nitric oxide NO and NGF as potential predictors of therapeutic outcomes in OAB patients undergoing LLLT. The observed increase in NO levels after LLLT shows that NO may play an important part in the therapeutic mechanism of LLLT. NO is known to modulate smooth muscle relaxation and bladder contractility, therefore increased production may help to alleviate OAB symptoms. The decrease in urine NGF levels in the LLLT group suggests that LLLT can control NGF resulting in enhanced bladder function and symptom relief. The present study reinforces the application of LLLT as a minimally invasive therapeutic approach for OAB, characterized by its superficial application and low discomfort levels during the procedure. Additionally, the observed levels of NO and urine NGF demonstrate promise as potential markers for gauging treatment effectiveness. This study paves the door for further research into the molecular mechanisms implicated in LLLT’s impacts on bladder function, as well as the creation of targeted therapies to improve patient outcomes in OAB care.

## Data Availability

The datasets used and/or analyzed during the current study are available from the corresponding author on reasonable request.
